# Overexpression of PTPRN Promotes Metastasis of Lung Adenocarcinoma and Suppresses NK Cell Cytotoxicity

**DOI:** 10.3389/fcell.2021.622018

**Published:** 2021-06-02

**Authors:** Xinyue Song, Xue Jiao, Han Yan, Lifeng Yu, Longyang Jiang, Ming Zhang, Lianze Chen, Mingyi Ju, Lin Wang, Qian Wei, Lin Zhao, Minjie Wei

**Affiliations:** ^1^Department of Pharmacology, School of Pharmacy, China Medical University, Shenyang, China; ^2^Liaoning Key Laboratory of Molecular Targeted Anti-Tumor Drug Development and Evaluation, China Medical University, Shenyang, China; ^3^Liaoning Cancer Immune Peptide Drug Engineering Technology Research Center, China Medical University, Shenyang, China; ^4^Key Laboratory of Precision Diagnosis and Treatment of Gastrointestinal Tumors, Ministry of Education, China Medical University, Shenyang, China; ^5^Shenyang Kangwei Medical Laboratory Analysis Co. LTD., Shenyang, China

**Keywords:** PTPRN, LUAD, EMT, tumor-infiltrating immune cells, NK cells

## Abstract

**Background:**

Lung adenocarcinoma (LUAD) is the most common diagnostic histologic subtype of non-small cell lung cancer, but the role of receptor-type tyrosine-protein phosphatase-like N (PTPRN) in LUAD has not been studied.

**Methods:**

We conducted a bioinformatic analysis to identify the expression of PTPRN on LUAD data from the Cancer Genome Atlas (TCGA) and the relationship between PTPRN and overall survival of LUAD patients. The effects of PTPRN on the migration ability of LUAD cells and the underlying mechanisms were investigated by *in vitro* and *in vivo* assays (i.e., wound healing assay, transwell assay, western blotting, xenograft model, and immunohistochemistry). Gene-set enrichment analysis and computational resource were used to analyze the correlation between PTPRN and different tumor-infiltrating immune cells (TIICs). Lactate dehydrogenase assay and Enzyme-linked immunosorbent assay were conducted to examine natural killer (NK) cell cytotoxicity.

**Results:**

In our study, we found that PTPRN was up-regulated in LUAD and related to metastasis of LUAD patients. Besides, PTPRN was correlated with poor prognosis in the TCGA-LUAD dataset. PTPRN overexpression promoted LUAD cell migration and the expression of EMT markers by influencing MEK/ERK and PI3K/AKT signaling. Moreover, PTPRN expression was significantly associated with TIICs, especially NK cells. A549 and H1299 cells overexpressed PTPRN inhibited NK cell cytotoxicity.

**Conclusion:**

Taken together, these findings demonstrated that PTPRN might be a potential and novel therapeutic target modulating antitumor immune response in treatment of LUAD.

## Introduction

Lung cancer, the main cause of cancer incidence and mortality, accounted for nearly a fifth of cancer deaths in 2018 (Bray et al., 2018). Besides, lung cancer is predicted to cause around a quarter of all cancer deaths in America in 2020 (Siegel et al., 2020). More than 80% of lung cancer cases are non-small cell lung cancer (NSCLC; Chen et al., 2014). Lung adenocarcinoma (LUAD), the most common histologic type of NSCLC, is responsible for almost 40% of deaths related to lung cancer (Spira and Ettinger, 2004; Fennell et al., 2016). Although there are many remarkable improvements in the treatment of LUAD, the overall survival (OS) of LUAD remains unsatisfactory. The 5-year survival rate of LUAD patients is no more than 20% and has not increased in the past few years (Kasinski and Slack, 2012; Song et al., 2018). Many patients die of its metastatic relapse after surgery (Li et al., 2013). Therefore, it is vital to discover new prognostic biomarkers and therapeutic strategies for patients who succumb to metastatic characteristics of LUAD.

Carcinogenesis is proved to be a long-term and sophisticated process that metastasis is a cardinal feature of OS (Glatzel-Plucinska et al., 2019). Epithelial–mesenchymal transition (EMT) plays a key role in embryonic development, fibrosis, and cancer metastasis (Pastushenko and Blanpain, 2019). EMT is regarded as the initial event related to metastasis of cancers. However, the mechanism of EMT is not fully elucidated. Thus, focusing on regulated molecules related to EMT might contribute to identify potential biomarkers of prognosis in cancers.

Receptor-type tyrosine-protein phosphatase-like N (PTPRN), also called islet cell autoantigen 512 (ICA512/IA-2), is a tyrosine phosphatase-like intrinsic membrane protein which participates in the biological process of insulin secretory granules in pancreatic islet β-cells (Toledo et al., 2019). It was reported to have a high expression in neuroendocrine cells (Takeyama et al., 2009; Gomi et al., 2013) and is related to diabetes (Steinbrenner et al., 2002). Several studies reported that PTPRN plays a potential role in solid tumors (Bauerschlag et al., 2011; Liang et al., 2018; Shergalis et al., 2018; Xu et al., 2018; Zhangyuan et al., 2018; Yin et al., 2019). PTPRN was overexpressed in a cluster of glioblastoma patients and indicated poor survival (Shergalis et al., 2018). Furthermore, Wen et al. and Xu et al. (2018) showed that PTPRN was a biomarker for predicting the prognosis of glioblastoma and could estimate the OS of glioblastoma patients (Yin et al., 2019). PTPRN was also an independent prognostic factor of hepatocellular carcinoma (Zhangyuan et al., 2018). Abnormal DNA hypermethylation of PTPRN was related to OS of patients with ovarian cancer (Bauerschlag et al., 2011). PTPRN mRNA and protein expression levels were increased in breast cancer cells under hypoxia (Liang et al., 2018). Although PTPRN could be a new marker for detecting neuroendocrine differentiation in lung cancer, the potential relationship between PTPRN and on EMT is still unclear (Xie et al., 1996).

The tumor microenvironment (TME) promotes immune escape which is regarded as one of cancer hallmarks (Hanahan and Weinberg, 2011). The relationship between immune cells in TME and cancer cells is continuous and participates from tumorigenesis initiation to tumor metastasis (Perez-Ruiz et al., 2020). A study indicated that EMT was closely related to TME (Li et al., 2020). NK cells are recruited to TME (Bald et al., 2020). NK cells play an important role in early tumors and metastasis elimination (Bald et al., 2020). The relationship between PTPRN, TME, and NK cells in LUAD needs to be further elucidated.

In the present study, we demonstrated that the expression of PTPRN was highly up-regulated in LUAD and associated with metastasis and poor OS of LUAD patients. PTPRN overexpression promoted LUAD cell metastasis capacity by influencing the MEK/ERK and PI3K/AKT pathways. Our study provided functional links between PTPRN and LUAD metastasis. Furthermore, our study reported that PTPRN influenced TME compositions and regulated NK cell regulation.

## Materials and Methods

### Publicly Available Data Analysis

Gene expression profiles and clinical data of LUAD patients were downloaded from the Cancer Genome Atlas (TCGA) database^1^. The samples of LUAD were divided into two groups according to the cutoff of the median PTPRN expression. The differences in N-cadherin, Slug, Snail, Twist, and Vimentin between the PTPRN low- and high-expression groups were analyzed. The Kaplan–Meier Plotter was used to analyze the correlation between PTPRN expression and survival time of lung cancer patients^2^.

The ESTIMATE algorithm by feat of R-package “estimate” was used to calculate immune scores that infer the proportion of stromal and immune cells of tumor samples (Yoshihara et al., 2013). It also exhibited three kinds of scores called ImmuneScore, StromalScore, and ESTIMATEScore, which were related to immune, stromal cells and the sum of them in TME.

### Evaluation of Tumor-Infiltrating Immune Cells

Computational resource (CIBERSORTx)^3^, an analytical tool based on gene expression profiles, predicts the ratios of member cell types in mixed cells (Newman et al., 2019). Thus, we can use this process to estimate tumor-infiltrating immune cells (TIICs). Immune response of 22 TIICs was measured to assess the association with PTPRN expression in LUAD. We established a dataset of gene expression by using standard annotation files and a defaulted signature matrix at 1000 permutations. CIBERSORTx predicted a *P*-value for deconvolution through Monte Carlo sampling to establish the confidence level in the outcomes. In order to analyze the impact of PTPRN in TME of LUAD, 534 tumor samples were utilized in the TCGA-LUAD dataset. We set *P*-value < 0.05 as the criterion to define the lymphocyte types affected by PTPRN.

Furthermore, the “correlation” module of GEPIA^4^ was used to determine the association between PTPRN expression and possible gene markers of TIICs. The gene markers included markers of CD8+ T cells, T cells (general), B cells, monocytes, tumor-associated macrophage (TAM), T-helper 1 (Th1) cells, T-helper 2 (Th2) cells, follicular helper T (Tfh) cells, T-helper 17 (Th17) cells, regulatory T cells (Treg), exhausted T cells, neutrophils, NK cells, and Mast cells. The correlation heat map was conducted to detect the relationship between every two different immune cells in 22 types of immune cells in TME.

### Gene-Set Enrichment Analysis

The TCGA-LUAD dataset with the gene sets (hallmark and C5 gene set v7.1 collections) was analyzed by Gene-set enrichment analysis (GSEA) in order to obtain biological processes enriched by PTPRN. The samples were divided into two groups: the top 50% of them were defined as the high PTPRN expression group, and the bottom 50% were defined as the low PTPRN expression group.

### NK Cell Isolation

Primary NK cells were purified from fresh peripheral blood samples from healthy volunteers. Peripheral blood mononuclear cells were isolated by RosetteSep^TM^ Human NK Cell Enrichment Cocktail (STEMCELL Technologies).

### Flow Cytometry Analysis

We stained NK cells with 5 μL/test FITC-CD3 (Biolegend) and 5 μL/test PE-CD56 (Biolegend) at 4°C for 30 min. NK cells were washed with PBS and suspended in 400 μL of PBS after staining. Flow cytometry analysis was performed on a BD FACSVia Flow Cytometer (BD Biosciences, United States).

### Cell Culture and Transfection

The H1299 and A549 lung cancer cell lines were obtained from the Institute of Biochemistry and Cell Biology at the Chinese Academy of Sciences (Shanghai, China). H1299 was maintained in the RPMI-1640 medium (HyClone) with 10% fetal bovine serum (FBS, PAA, Germany), 100 mg/ml penicillin (Invitrogen), and 100 U/mL streptomycin (Invitrogen). A549 was maintained in F-12K medium (Gibco) with 10% fetal bovine serum (FBS, PAA, Germany), 100 mg/ml penicillin (Invitrogen), and 100 U/mL streptomycin (Invitrogen). Purified NK cells were cultured in NK cell culture medium (Cell Science and Technology Institute Inc.). NK cells and H1299 and A549 cell lines were cultured at 37°C in a 5% CO2 and 95% air incubator. PTPRN-pcDNA3.1 and pcDNA3.1 vectors were purchased from Sangon Co., Ltd. (Shanghai, China). The empty vector was obtained from Sangon. The cells were transfected with plasmids using Lipofectamine 3000 (Invitrogen) according to the manufacturer’s protocol. To obtain LLC cells which overexpressed PTPRN stably, LLC cells were electroporated with the BIO-RAD GenePulser Xcell system (BIO-RAD, United States). The condition of different transfection parameters (pulse settings) was tested in order to optimize the parameters (Ingegnere et al., 2019). The electroporation pulse voltage and width were adjusted. We found that the optimal condition of voltage pulse was 248 V and the optimal condition of pulse width was 30 ms.

### Wound-Healing Assay

For detecting cell migration, wound assay was conducted; A549 and H1299 cells with transection of each group were seeded into six-well plates. When cells reached around 90% confluency, a pipette tip was used vertically to make scratches on the cell layer. Cells were washed with PBS to clear scratched cell debris and cultured with a serum-free medium in the incubator. Cells were observed and photographed using a microscope (Nikon, Japan) after cultured for 0 h and 24 h.

### Transwell Migration Assay

Cell migration was assessed in a 24-well transwell chamber (3422, Corning, United States) according to the manufacturer’s protocol. H1299 and A549 cells were seeded at a density of 4 × 10^4^ cells/well in the upper chambers with a serum-free RPMI-1640 medium. The lower chambers contained the RPMI-1640 medium with 20% FBS. After 24 h, cells which migrated out of the inserts were immobilized by methanol and stained with 0.1% crystal violet (Solarbio, China). Cells were observed using the microscope and photographed (magnification, ×100). Cells were counted by five fields per well at random.

### Western Blotting

Cells were lysed by radioimmunoprecipitation assay lysate buffer (Beyotime, Jiangsu, China) on ice for 30 min. The supernatants of cells were collected. Bicinchoninic acid protein quantitation (Takara, Japan) was used to determine protein concentrations. Proteins from cell lysate were transferred to PVDF membranes (Millipore, Bedford, MA, United States) after SDS-PAGE electrophoresis. The primary and secondary antibodies are shown in Table 1.

**TABLE 1:** Antibodies used for WB and IHC.

**Antibody**	**Company/provider**	**Dilution ratio**
Anti-human PTPRN	Proteintech	1:1000
Anti-human Vimentin	Cell signaling technology	1:1000
Anti-human E-cadherin	Cell signaling technology	1:1000
Anti-human N-cadherin	Cell signaling technology	1:1000
Anti-human β-actin	BOSTER	1:1000
Anti-human ERK1/2	Cell signaling technology	1:1000
Anti-human p-ERK1/2	Cell signaling technology	1:1000
Anti-human AKT	Proteintech	1:1000
Anti-human p-ERK1/2	Proteintech	1:1000
Anti-human Cyclin D1	Cell signaling technology	1:1000
Goat anti-mouse IgG	Absin bioscience Inc	1:10000
Goat anti-rabbit IgG	Absin bioscience Inc	1:10000
Anti-human PTPRN (for IHC)	Proteintech	1:100
Anti-human Vimentin (for IHC)	Cell signaling technology	1:100
Anti-mouse KLRA1 (for IHC)	Bioss	1:100

### IHC and Quantification Analysis

Mouse tumor sections and LUAD tissue samples were dewaxed by xylene and rehydrated by graded alcohol series. We used citrate buffer (pH = 6.0) to retrieve the antigen under high pressure. We used Ultra-sensitive S-P kit (Maixin-Bio, China) to block the endogenous peroxidase activity of sections. The sections were incubated with primary antibodies (shown in Table 1) at 4°C overnight. Then, sections were incubated with a secondary antibody. DAB reagent was used to visualize the sections.

Every slide was examined five times, and cells were observed at 400 × magnification. The expression of PTPRN was estimated by staining intensity and percentage of positive cells. Staining intensity was defined as 0 (negative), 1 (weak), 2 (moderate), and 3 (strong). The percentage of positive cells was defined as 0 (<5%), 1 (6–25%), 2 (26–50%), 3 (51–75%), and 4 (>76%). The final staining score of Immunohistochemistry (IHC) was calculated by multiplying the staining intensity levels with the positive percentage staining scores (Zhao et al., 2018).

### Lactate Dehydrogenase Cytotoxicity Assay

The cytotoxicity of NK cells was conducted by lactate dehydrogenase (LDH) cytotoxicity assay (Beyotime, China), according to the manufacturer’s instructions. Briefly, we collected cell supernatants which cocultured NK cells and A549 and H1299 cells for 4 h. Then, we performed LDH assay. The formula of cytotoxicity was calculated: Cytotoxicity (%) = [(OD_*Experimental*_ value–OD_*Spontaneous*_ value)/(OD_*Maximum*_ value–OD_*Spontaneous*_ value)] × 100 (Bian et al., 2020).

### Enzyme-Linked Immunosorbent Assay

IFN-γ and TNF-α in cell supernatants were detected using human IFN-γ precoated Enzyme-linked immunosorbent assay (ELISA) kit and human TNF-α precoated ELISA kit (DAKEWE, China) according to the manufacturer’s instructions. A549 and H1299 cells were seeded in 96-well plates at a density of 1.0 × 10^4^ cells per well. NK cells were added into the 96-well plates at a density of 1.0 × 104 cells per well. After 4 h of incubation, cell supernatants were collected to perform ELISA assay. Absorbance was read at Infinite 200 PRO microplate reader (TECAN, Switzerland).

### Xenograft Model

To study the influence of PTPRN in LUAD, LLC cells (1 × 10^6^) were suspended in 100 μL of PBS and injected into mammary fat pads of 4- to 5-week-old BALB/c(nu/nu) mice (Hua Fukang Biological Technologies Inc, Beijing, China). Mice were randomized into the following four groups (*n* = 6 per group): NC-cDNA, PTPRN-cDNA, NC-cDNA with TGF-β1, and PTPRN-cDNA with TGF-β1. TGF-β1 (Novoprotein; 4 μg/kg bodyweight) was injected intraperitoneally (i.p.) every 5th day after cell inoculation as previously described to establish TGF-β-induced cancer cell invasion (Wang et al., 2018). The tumor diameter and weights of mice were measured every other day. Tumors were removed from xenograft models and weighed until tumors appeared to collapse. Tumor volume (mm^3^) was measured using a digital caliper and calculated according to the following equation: (width)^2^ × (length/2). All mice were bred at pathogen-free conditions in the Animal Centre of China Medical University. All animal studies were performed according to the National Institute of Health Guide for the Care and Use of Laboratory Animals.

### Statistical Analysis

Quantitative data were expressed as the means ± SD of at least three independent experiments. GraphPad Prism 7.0 (GraphPad Software, La Jolla, CA, United States) was used to evaluate all experimental values. Student’s independent *t* test was used to perform statistical analysis between two experimental groups, while analysis of variance (ANOVA) was used to perform analyses among three experimental groups. Pearson’s correlation coefficient was used to analyze the correlation between PTPRN and ImmuneScore, StromalScore, and ESTIMATEScore. *P* value < 0.05 was considered statistically significant in all cases.

## Results

### Detection of PTPRN Protein Expression Is Analyzed by IHC

**FIGURE 1:**
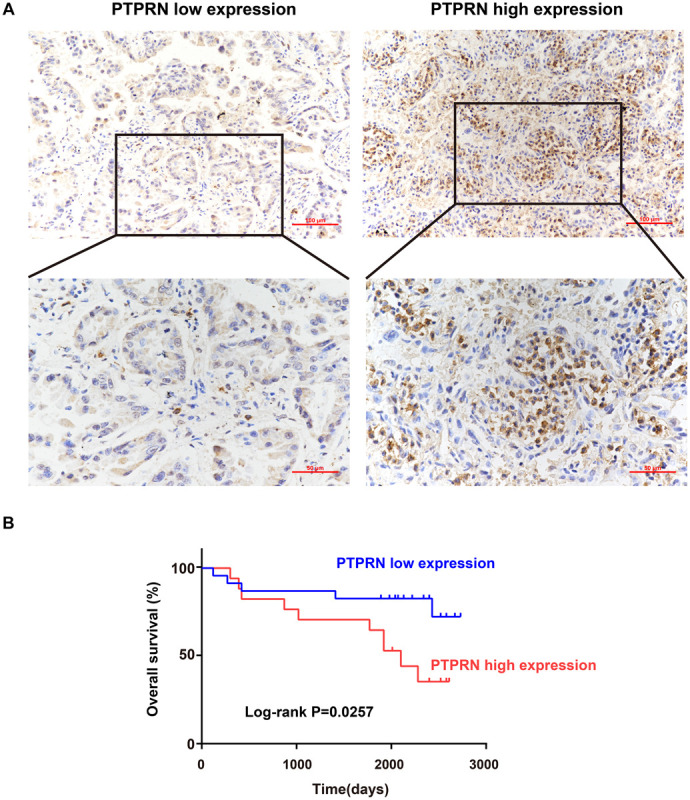
Association of PTPRN expression with OS by IHC. **(A)** IHC is used to stain for PTPRN in tumor samples from 40 LUAD patients. Representative IHC pictures of the high and low PTPRN expression groups are shown at ×20 and ×40 magnification (scale bars, 100 μm and 50 μm, respectively). **(B)** Kaplan–Meier survival analysis of 40 LUAD patients shows that the PTPRN high expression is related to unfavorable prognosis.

Sectioning experiments of 40 LUAD clinical samples have been done to elucidate the relationship between PTPRN and OS. Representative results for PTPRN protein in LUAD tissue are shown in Figure 1A. According to the ROC curve (AUC = 0.727, *P* = 0.0019), IHC scores of PTPRN were classified as low expression group (≤2) or high expression group (>2; data not shown). For survival analysis, the LUAD patients were divided into two groups with low or high PTPRN expression by IHC scores. The Kaplan–Meier survival curve demonstrated that LUAD with PTPRN high expression had an unfavorable OS (*P* = 0.0257; Figure 1B).

### PTPRN Influences LUAD Cell Migration *in vitro*

**FIGURE 2:**
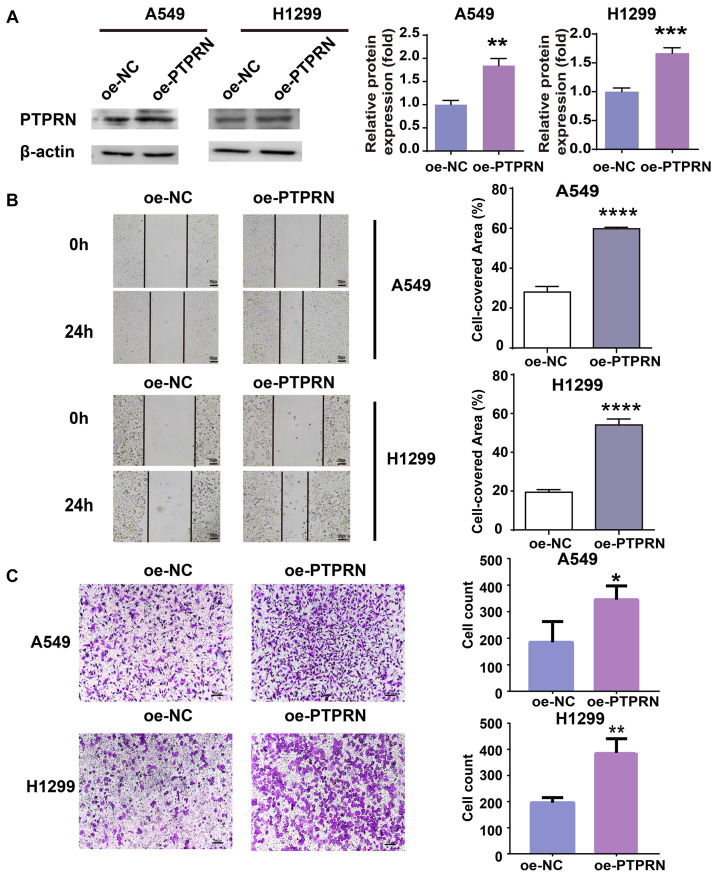
Effect of overexpression of PTPRN expression on migration of LUAD cells. **(A)** The expression of PTPRN in A549 and H1299 cells after transfection of the PTPRN plasmid. **(B)** The effect on migration of A549- and H1299-overexpressed PTPRN is detected by wound healing assay. **(C)** The effect on migration of A549- and H1299-overexpressed PTPRN is identified by transwell migration assay. Original magnification, ×100. Scale bars, 100 μm. *P < 0.05, **P < 0.01, ***P < 0.001, and ****P < 0.0001. oe-NC, negative control; and oe-PTPRN, PTPRN overexpression group.

To identify the effect of PTPRN on migration of LUAD cells, PTPRN was overexpressed in A549 and H1299 with low expression of PTPRN. The overexpression efficiency of PTPRN was evaluated by western blot. After transfection with PTPRN, PTPRN expression was upregulated in comparison with the negative control group (Figure 2A). The migration ability of the control group and PTPRN-overexpressing cells was examined. The wound-healing assay significantly revealed that the cell-covered area was increased in the PTPRN-overexpressed group compared with the negative control group in Figure 2B. Furthermore, we found that the number of migrated cells was increased in the PTPRN-overexpressed group compared with the control group in transwell migration assay (Figure 2C).

### PTPRN Is Related to Metastasis of LUAD Patients

**FIGURE 3:**
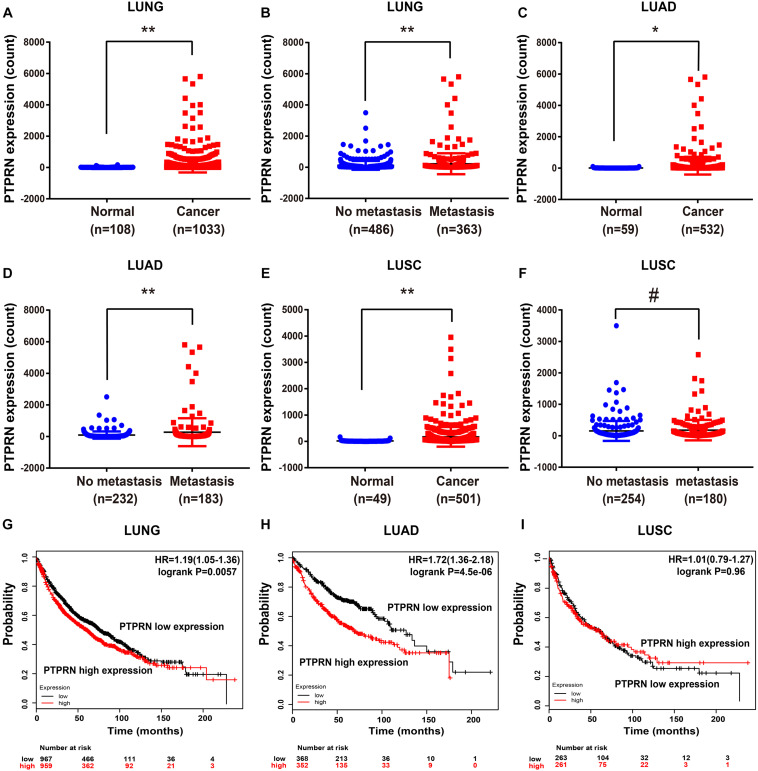
PTPRN expression in normal and lung cancer tissues in TCGA and survival time analyzed by Kaplan–Meier Plotter. **(A,B)** mRNA expression of PTPRN is analyzed in lung cancer in the TCGA-LUNG database. **(C,D)** mRNA expression of PTPRN is analyzed in LUAD in the TCGA-LUAD database. **(E,F)** mRNA expression of PTPRN is analyzed in LUSC in the TCGA database. **(G)** Kaplan–Meier survival analysis of lung cancer patients indicates that the decrease in PTPRN expression is correlated with favorable prognosis (*P* = 0.0057). **(H)** Kaplan–Meier survival analysis of LUAD patients shows that the decrease in PTPRN expression is correlated with favorable prognosis (*P* < 0.0001). **(I)** Kaplan–Meier survival analysis indicates that there is no correlation between PTPRN expression and prognosis of LUSC patients (*P* = 0.96). *P < 0.05, **P < 0.01. ^#^*P* > 0.05.

To elucidate whether PTPRN affected metastasis of lung cancer, we evaluated the expression level of PTPRN in TCGA datasets. The TCGA RNA Seq data demonstrated that PTPRN was significantly overexpressed in lung cancer compared with noncancerous tissue samples (Figure 3A). Besides, PTPRN was highly expressed in NSCLC patients with distant metastasis and lymph-node metastasis compared with non-metastatic NSCLC patients (Figure 3B). PTPRN expression in LUAD was distinctively higher than that of normal tissues (Figure 3C). PTPRN was also highly expressed in metastatic LUAD patients compared with non-metastatic LUAD patients (Figure 3D). Meanwhile, PTPRN was also significantly expressed in LUSC compared with normal tissues (Figure 3E). There were no differences in PTPRN expression between metastatic LUSC patients and non-metastatic patients (Figure 3F). Then, we performed bioinformatic analysis of data mining with the Kaplan–Meier plotter. We found that PTPRN high expression was relevant to a shorter OS in NSCLC patients and LUAD patients (Figures 3G,H). However, OS in LUSC patients showed no correlation between high PTPRN expression and low PTPRN expression (Figure 3I). These results demonstrated that PTPRN was related to metastasis of LUAD and affected OS of LUAD patients, not in LUSC patients.

### High PTPRN Expression Was an Independent Prognostic Factor for Poor OS in LUAD Patients

In order to elucidate whether PTPRN was involved in LUAD development, we analyzed the relationship between the expression of PTPRN and clinical pathology factors. Univariate analysis (Table 2) revealed that the advanced pathologic stage (III–IV), tumor stage (T3–T4), regional lymph node (N1–N3), metastasis (M1), and high PTPRN expression were relevant to remarkably shorter OS in LUAD patients (Table 2). Then, the multivariate analysis verified that the high expression of PTPRN was an independent prognostic factor for poor OS (Table 2).

**TABLE 2:** Univariate and multivariate analyses of OS in patients with LUAD in TCGA.

	**Univariate analysis**			**Multivariate analysis**		
	**HR**	***p* value**	**95% CI**	**HR**	***p* value**	**95% CI**
**Age**						
>66 versus ≤66	1.218	0.268	0.859–1.726			
Gender						
Male versus female	1.076	0.679	0.760–1.523			
Smoking history						
2/3/4/5 versus 1	1.075	0.772	0.659–1.753			
**Pathologic stage**						
III–IV versus I–II	2.503	<0.001	1.741–3.599	1.009	0.979	0.514–1.980
Tumor stage						
T3–T4 versus T1–T2	2.586	<0.001	1.682–3.977	2.194	0.002	1.344–3.583
Regional lymph node						
N1–N3 versus N0	2.438	<0.001	1.719–3.457	2.104	0.015	1.158–3.823
Metastasis						
M1 versus M0	1.878	0.032	1.056–3.341	1.502	0.188	0.820–2.750
**PTPRN**						
High versus Low	1.710	0.003	1.200–2.436	1.470	0.036	1.026–2.107

### PTPRN Is Associated With EMT of LUAD Through MEK/ERK and PI3K/AKT Signaling Pathways

**FIGURE 4:**
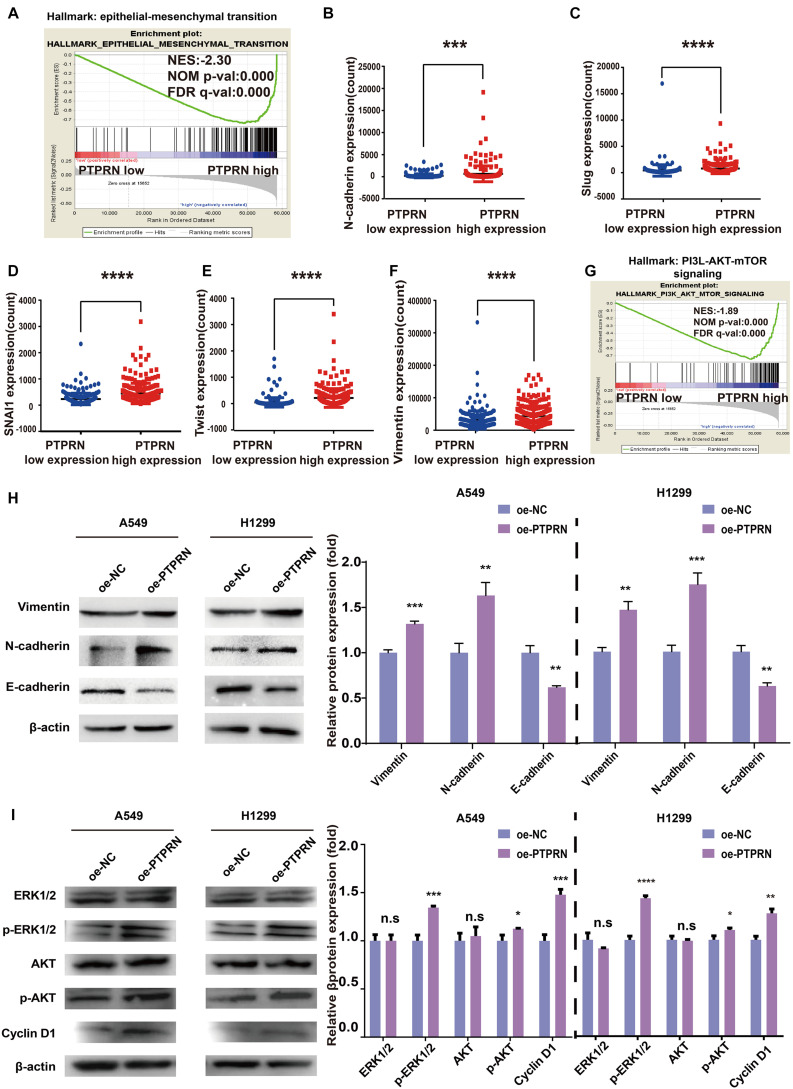
Bioinformatic analysis and western blot indicating that PTPRN expression is related to EMT. **(A)** The result of GSEA shows that EMT is enriched in the high-PTPRN expression group. PTPRN promotes mRNA expression levels of EMT markers such as **(B)** N-cadherin, **(C)** Slug, **(D)** Snail, **(E)** Twist, and **(F)** Vimentin from RNA sequencing data from the TCGA-LUAD database. **(G)** The PI3K/AKT/mTOR signaling pathway was enriched in the PTPRN high-expression group by GSEA. **(H)** Left: Expression of EMT markers (Vimentin, N-cadherin, and E-cadherin) is detected in PTPRN-overexpressing A549 and H1299 cells by western blotting. Right: Densitometric analysis of protein expression. **(I)** Left: Proteins in MAPK/ERK and PI3K/AKT pathways are detected in PTPRN-overexpressing A549 and H1299 cells by western blotting. Right: Densitometric analysis of protein expression. *P < 0.05, **P < 0.01, ***P < 0.001, and ****P < 0.0001.

To illustrate how PTPRN influenced the metastatic phenotype of LUAD, LUAD samples of TCGA were divided into PTPRN high and low expression groups by the median of PTPRN expression. GSEA was conducted to choose significant enriched pathways according to the normalized enrichment score (NES). Then, we found that PTPRN was related to EMT (Figure 4A and Table 3). EMT is regarded as a vital step in metastasis progression and promotes cancer cell migration and invasion. Then, we analyzed the relationship between PTPRN and EMT markers. The expressions of EMT markers including N-cadherin, Slug, Snail, Twist, and Vimentin were significantly higher in PTPRN-high patients than PTPRN-low patients (Figures 4B–F).

**TABLE 3:** A correlation analysis using the “Correlation” module of GEPIA.

**Description**	**Gene markers**	**LUAD**			
		**Tumor**		**Normal**	
		***R***	***P***	***R***	***P***

CD8+ T cell	CD8A	-0.018	0.7	-0.27	0.04
	CD8B	0.0036	0.94	-0.22	0.098
T cell (general)	CD3D	-0.055	0.22	-0.12	0.35
	CD3E	-0.091	0.046	-0.12	0.38
	CD2	-0.011	0.013	0.018	0.89
B cell	CD19	0.052	0.26	0.053	0.69
	CD27	0.027	0.56	-0.15	0.26
Monocyte	CD86	0.085	0.061	-0.19	0.16
	CD115	0.062	0.18	0.051	0.70
TAM	CCL2	0.15	0.00076	-0.065	0.63
	CD68	0.16	0.00046	-0.19	0.16
	IL-10	0.079	0.085	-0.11	0.39
Th1	IFN-γ (IFNG)	0.059	0.19	0.17	0.20
	STAT1	0.18	6.3e-05	0.17	0.21
	T-bet (TBX21)	-0.058	0.21	0.14	0.29
	TNF-α (TNF)	0.022	0.62	0.15	0.25
Th2	STAT6	-0.24	6e-08	0.062	0.64
	GATA3	0.0014	0.97	-0.01	0.94
	IL13	-0.0085	0.85	-0.038	0.77
	STAT5A	-0.051	0.27	0.054	0.69
Tfh	BCL6	-0.057	0.21	0.044	0.74
Th17	STAT3	0.0018	0.97	0.078	0.56
	IL17A	-0.028	0.54	0.014	0.92
Treg	CCR8	0.015	0.75	0.16	0.22
	STAT5B	0.013	0.78	0.14	0.27
	FOXP3	0.043	0.35	0.15	0.27
T cell exhaustion	LAG3	0.11	0.014	0.19	0.16
	CTLA4	0.021	0.64	0.044	0.74
	PD-1 (PDCD1)	0.078	0.085	-0.099	0.46
	TIM-3	0.059	0.19	-0.21	0.10
Neutrophils	CD11b	0.027	0.55	-0.15	0.26
	CCR7	-0.13	0.003	0.013	0.92
Natural killer cell	KIR2DL1	0.088	0.052	0.037	0.78
	KIR2DL3	0.1	0.022	0.039	0.77
	KIR2DL4	0.24	7e-08	-0.11	0.42
	KIR3DL1	0.05	0.27	-0.0078	0.95
	KIR3DL2	0.077	0.09	0.0047	0.97
	KIR3DL3	0.13	0.0036	-0.084	0.53
	CD56	0.14	0.0026	0.15	0.24
Mast cell	TPSB2	-0.094	0.039	-0.05	0.7
	TPSAB1	-0.08	0.079	0.033	0.8
	CPA3	-0.092	0.044	-0.037	0.78
	MS4A2	-0.15	0.0082	-0.0059	0.96
	HDC	-0.099	0.029	-0.088	0.51

*Tumor, correlation analysis in LUAD tumor tissue of TCGA. Normal, correlation analysis in LUAD normal tissue of TCGA.*

Furthermore, we also found that PTPRN was related to the PI3K/AKT/mTOR signaling pathway by GSEA (Figure 4G). Western blot assay showed that Vimentin and N-cadherin expression levels were increased in PTPRN-overexpressing A549 and H1299 cells (Figure 4H). However, E-cadherin expression level was decreased in PTPRN-overexpressing A549 and H1299 cells (Figure 4H). The protein expression of the phosphorylated ERK1/2, AKT, and cell cycle-related protein Cyclin D1 was increased by overexpressing PTPRN in A549 and H1299 cells (Figure 4I). However, PTPRN barely affected the total protein expression of ERK1/2 and AKT (Figure 4I). These results above added increasing evidence that PTPRN was related to EMT characteristics and regulated MEK/ERK and PI3K/AKT signaling pathways.

### PTPRN Is Related to Immune Infiltrates

**FIGURE 5:**
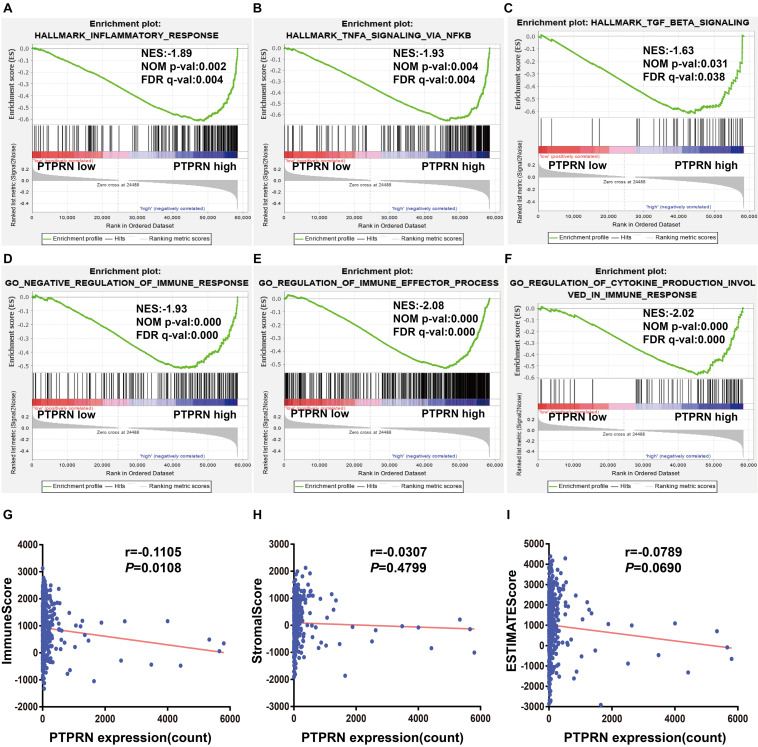
The association between PTPRN expression and immune response by GSEA in the TCGA-LUAD dataset. GSEA shows that **(A)** inflammatory response, **(B)** TNFα signaling via NFκB, **(C)** TGFβ signaling, **(D)** negative regulation of immune response, **(E)** regulation of immune effector process, and **(F)** regulation production involved in immune response are enriched in high PTPRN expression. **(G)** PTPRN expression is negatively related to ImmuneScore. **(H)** There is no correlation between PTPRN expression and StromalScore. **(I)** PTPRN expression is not related to ESTIMATEScore.

Gene-set enrichment analysis also found that high PTPRN expression was enriched in inflammatory response, TNFα signaling via NF-κB and TGFβ signaling pathways (Figures 5A–C and Table 3), indicating that PTPRN might be associated with immune response in LUAD to promote EMT. Furthermore, we demonstrated several immune-related pathways by GSEA (Figures 5D–F and Table 4), including negative regulation of immune response, regulation of immune effector process, and regulation production involved in immune response. These results showed that PTPRN was involved in immune response. Higher scores calculated in ImmuneScore, StromalScore, and ESTIMATEScore represented a higher proportion of immune and stromal components in TME. The previous study showed that these increased scores of LUAD inferred favorable survival of LUAD patients (Bi et al., 2020). PTPRN was negatively correlated with ImmuneScore (*P* = 0.0108, Figure 5G). However, PTPRN expression had no correlation with StromalScore and ESTIMATEScore (*P* = 0.4799 and *P* = 0.0690, respectively, Figures 5H,I). These results reminded us that PTPRN might influence immune cell infiltration.

**TABLE 4:** Gene set enriched in LUAD samples with high-expression PTPRN.

**MsigDB collection**	**Gene set name**	**NES**	**NOM *p*-val**	**FDR *q*-val**
h.all.v7.1.symbols.gmt	HALLMARK_EPITHELIAL_MESENCHYMAL_TRANSITION	−2.30	0.000	0.000
	HALLMARK_PI3K_AKT_mTOR	−1.89	0.000	0.000
	HALLMARK_TNFA_SIGNALING_VIA_NFKB	−1.93	0.004	0.004
	HALLMARK_INFLAMMATORY_RESPONSE	−1.89	0.002	0.004
	HALLMARK_TGF_BETA_SIGNALING	−1.63	0.031	0.038
c5.all.v7.1.symbols.gmt	GO_NEGATIVE_REGULATION_OF_IMMUNE_RESPONSE	−1.93	0.000	0.000
	GO_REGULATION_OF_IMMUNE_EFFECTOR_PROCESS	−2.08	0.000	0.000
	GO_REGULATION_OF_CYTOKINE_PRODUCTION_INVOLVED_IN_IMMUNE_RESPONSE	−2.02	0.000	0.000

*NES: normalized enrichment score; NOM: nominal; FDR: false discovery rate.*

### The Expression of PTPRN Is Correlated With Proportion of Immune Infiltrating Cells

**FIGURE 6:**
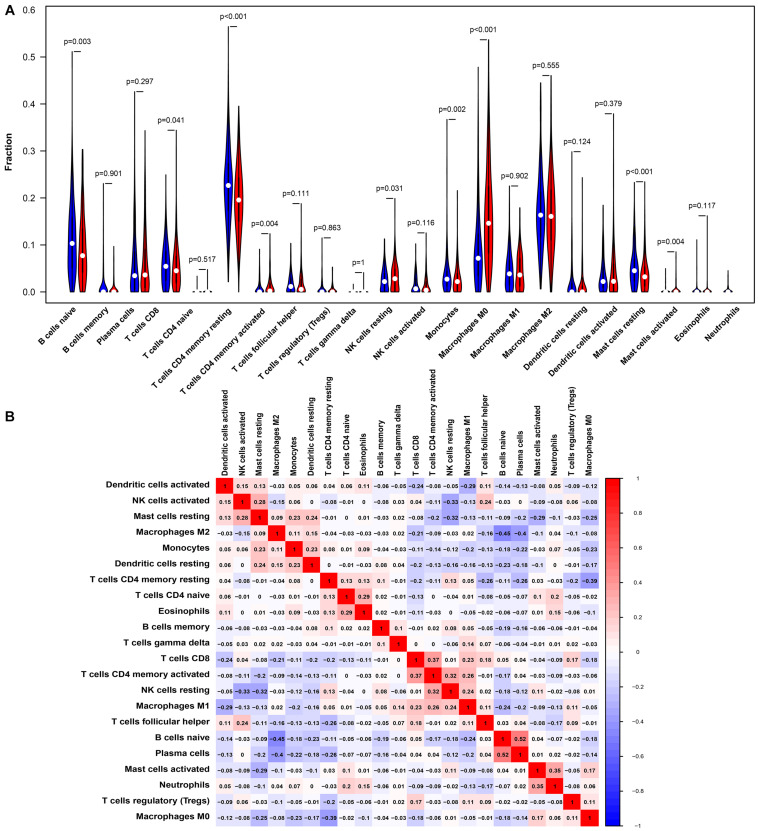
PTPRN-related immune infiltration alteration. **(A)** The fractions of activated memory CD4 T cells, resting NK cells, M0 macrophages, activated mast cells, and neutrophils are higher in the high PTPRN expression group. The proportions of naive B cells, CD8 T cells, resting memory T cells, monocytes, and resting mast cells are lower in high-expression PTPRN. **(B)** The ratios of different types of immune cells are correlated in the tumor microenvironment.

Studies have shown that tumor-infiltrating lymphocytes are an independent factor predicting survival in cancer patients (Azimi et al., 2012; Wang et al., 2020). Different cancers had various immune infiltrations, which affects cancer cell proliferation and metastasis. Therefore, we further investigated the correlation between PTPRN expression and immune infiltrating cells in the microenvironment of LUAD patients. The data of 534 LUAD tumor tissue samples were downloaded from TCGA and divided into high and low expression by the median expression of PTPRN. We used an established CIBERSORTx to explore the gene expression profiles of LUAD samples to infer the different fractions of 22 types of immune cells between high expression group and low expression group. Figure 6 shows the results of 22 immune cell subtypes. As shown in Figure 6A, immune cells such as naive B cells, T cells CD8, resting memory T cells CD4, activated memory T cells CD4, resting NK cells, monocytes, M0 macrophages, resting mast cells, activated mast cells, and neutrophils were affected by PTPRN expression. The fractions of activated memory CD4 T cells (*P* = 0.002), resting NK cells (*P* = 0.024), macrophages M0 (*P* < 0.001), activated mast cells (*P* = 0.004), and neutrophils (*P* < 0.001) were up-regulated in the high-PTPRN expression group. Meanwhile, the proportions of naive B cells (*P* = 0.004), CD8 T cells (*P* = 0.05), resting memory T cells (*P* < 0.001), monocytes (*P* = 0.001), and resting mast cells (*P* < 0.001) were down-regulated in high-expression PTPRN. Then, relationships between 22 immune cell subsets were analyzed. The correlation heat map (Figure 6B) reflected that there were weak and moderate correlations between the proportions of different TIICs subpopulations.

The correlation between PTPRN expression and different immune cell type markers was further investigated in LUAD through the “Correlation” module of GEPIA. We used Spearman correlation to evaluate the correlation coefficient. Our study reminded us that gene markers affected by PTPRN expression included CD3E and CD2 of T cell (general); CCL2 and CD68 of TAM; STAT1 of Th1; STAT6 of Th2; LAG3 of T cell exhaustion; CCR7 of neutrophils; KIR2DL3, KIR2DL4, KIR3DL3, and CD56 of NK cell; and TPSB2, CPA3, MS4A2, and HDC of mast cell (Table 4). Results between PTPRN expression and markers of T cells, macrophages, neutrophils, mast cells, and NK cells were similar to the results of CIBERSORTx. According to the results of CIBERSORTx and GEPIA, these findings indicated that PTPRN might play a key role in regulating T cells, macrophage, neutrophils, mast cells, and NK cells of the tumor-infiltrating immune environment. According to GEPIA analysis, PTPRN was associated with NK cell inhibitory markers, indicating that PTPRN might influence NK cell activation.

### PTPRN Is Related to Inhibitory Markers of NK Cells and Inhibits NK Cell Cytotoxicity

**FIGURE 7:**
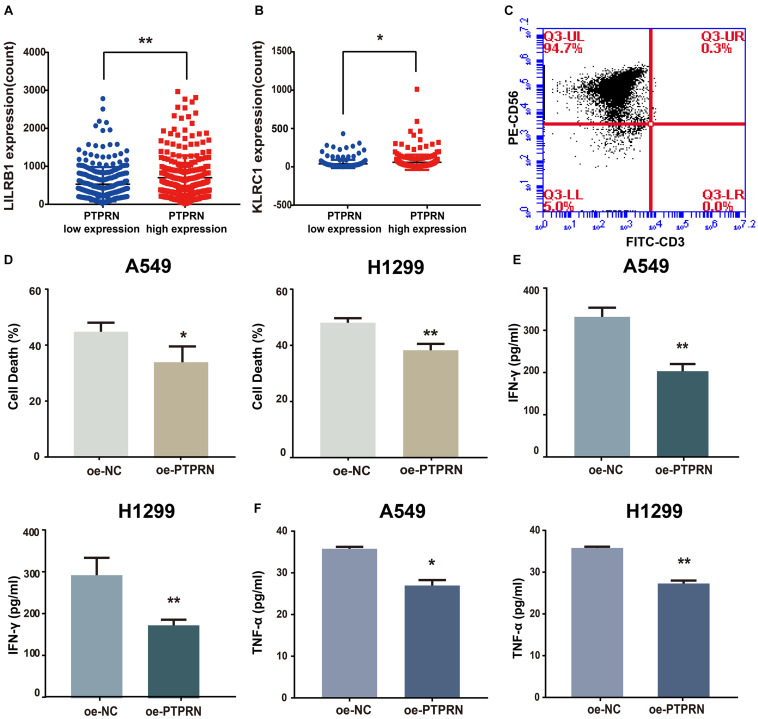
The potential affection of PTPRN on NK cells. PTPRN affects mRNA expression levels of NK cell inhibitory receptors such as **(A)** LILRB1 and **(B)** KLRC1 from RNA-sequencing data from the TCGA-LUAD database. **(C)** Percentages of CD3^–^ CD56^+^ cells of NK cells are determined by flow cytometry. **(D)** NK cytotoxicity assay is conducted with A549 target cells that overexpressed PTPRN on Day 14. The effector-to-target (E: T) ratio is 5:1. A similar assay is conducted with H1299 target cells on Day 14. The E:T ratio is 5:1. NK cells are treated with LUAD cells that overexpressed PTPRN. IFN-γ **(E)** and TNF-α **(F)** secretion is analyzed using ELISA. Data are presented as the mean ± SD of three independent experiments performed in triplicate. *P < 0.05, **P < 0.01.

To identify how PTPRN influences NK cells, we analyzed the relationship between PTPRN and NK cell inhibitory receptors in the TCGA-LUAD dataset. We found that NK cell inhibitory markers such as LIRB1 and KLRC1 were expressed higher in the PTPRN high-expression group compared with the PTPRN low-expression group (Figures 7A,B). Peripheral blood NK cells were isolated from healthy donors. In peripheral blood, human NK cells accounted for approximately 10–15% of cells. NK cells were identified according to the CD3^–^CD56^+^ profile. NK cells were determined by flow cytometry on Day 14. We obtained 90.97 ± 2.55% CD3^–^CD56^+^ NK cells after purification of NK cells (Figure 7C). NK cell cytotoxic activity was measured by LDH assay. As shown in Figure 7D, stimulated NK cells decreased cytotoxicity when A549 and H1299 overexpressed PTPRN. Furthermore, we found that IFN-γ and TNF-α levels in cell culture supernatant were reduced when A549 and H1299 were overexpressed PTPRN (Figures 7E,F). These results suggested that PTPRN overexpression in LUAD cells might inhibit NK cell cytotoxicity.

### PTPRN Influences EMT and NK Cell Inhibitory Marker *in vivo*

**FIGURE 8:**
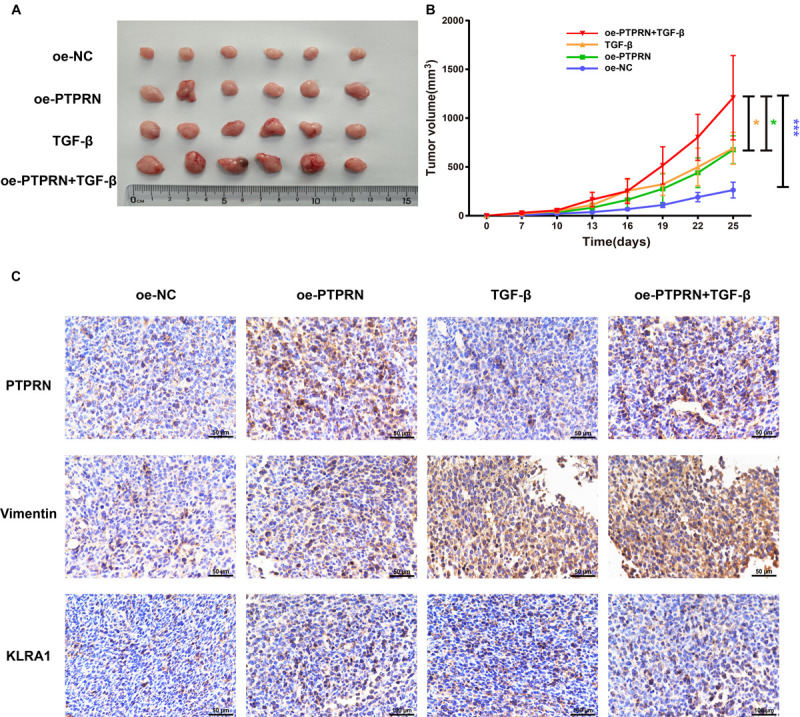
The effect of PTPRN on EMT and NK cell inactivation *in vivo.* **(A)** Images of oe-PTPRN LLC tumor tissues. **(B)** Average tumor volumes are measured in xenograft mice every 2 days. **(C)** IHC analysis of vimentin and KLRA1 protein levels in tumor tissues formed from PTPRN-overexpressing cells or control cells. Original magnification, ×400. Scale bars, 50 μm. Data are presented as the mean ± SD of three independent experiments performed in triplicate. *P < 0.05, ***P < 0.001. oe-NC, NC-cDNA group; oe-PTPRN, PTPRN-cDNA group; TGF-β, NC-cDNA with the TGF-β group; and oe-PTPRN+ TGF-β, PTPRN-cDNA with the TGF-β group.

To identify the effect of PTPRN on EMT and NK cell inhibition, LLC cells transfected with NC-cDNA or PTPRN-cDNA were subcutaneously injected into nude mice. Then, TGF-β1 was injected i.p. post cell inoculation. A study showed that TGF-β1 is one of the strongest inducers of EMT (Chen et al., 2017). Tumor size was significantly increased in the PTPRN overexpression group compared with negative control. Besides, TGF-β1 had the same effect on increasing tumor size (Figures 8A,B). Consistent with *in vitro* experiments, PTPRN promoted the expression of vimentin and KLRA1 (NK cells inhibitory marker of mice) detected by IHC (Orr and Lanier, 2011). TGF-β1 had the same effect on increasing vimentin expression compared with PTPRN (Figure 8C). Furthermore, TGF-β1 and PTPRN had a synergistic effect on the increase in vimentin expression (Figure 8C). These results indicated that PTPRN promoted EMT and NK cell inactivation *in vivo*.

## Discussion

Protein phosphatase-like N was a membrane protein associated with diabetes. Mziaut and colleagues found that the depletion of ICA512 reduced insulin release stimulated by glucose through downregulating F-actin modifier villin in pancreatic islet β-cells (Mziaut et al., 2016). Besides, PTPRN was reported to be overexpressed in several cancers such as glioblastoma (Xu et al., 2018; Yin et al., 2019) and hepatocellular carcinoma (Zhangyuan et al., 2018). In our study, data mining by the TCGA dataset was performed to demonstrate that PTPRN is up-regulated in LUAD tissues and high PTPRN expression is related to poor prognosis in LUAD patients. Surprisingly, we also found that high PTPRN expression is associated with metastasis of LUAD patients. The molecular mechanism of PTPRN promoting LUAD metastasis is still unclear.

Cancer metastasis is a complex process where cancer cells migrate from the primary site to another tissue for colonization (Lambert et al., 2017; Zada et al., 2019), causing most cancer-related deaths (Mehlen and Puisieux, 2006). Metastasis is divided into four steps: (1) detachment from the primary tumor; (2) blood migration; (3) stromal tissue invasions; and (4) proliferation in distant location (Eccles and Welch, 2007). EMT with characteristics of loss of epithelial properties and increase of mesenchymal properties is crucial to initiation of metastasis (Nieto et al., 2016; Zada et al., 2019).

Furthermore, GSEA found that PTPRN was related to EMT. The TCGA-LUAD dataset showed that PTPRN is associated with EMT markers such as N-cadherin, Slug. Then, we transfected PTPRN into A549 and H1299 cells. The cells with PTPRN high expression expressed more EMT markers such as N-cadherin at mRNA and protein levels. Wound healing assay and transwell cell assay were conducted, suggesting that PTPRN promotes LUAD cell invasion and migration by regulating the EMT pathway. Then, our results indicated that the expressions of Vimentin, N-cadherin, p-ERK, and p-AKT were up-regulated in the PTPRN overexpression group compared with the negative control group. Some studies have shown that EMT was related to the MEK/ERK and PI3K/AKT signaling pathways (Cao et al., 2019; Liu et al., 2020; Schafer et al., 2021).

The immune cells in TME are vital to the development of tumors and could be an indicator of prognostic and diagnostic assessment of cancer patients (Stankovic et al., 2018). Then, we discussed whether PTPRN influences immune infiltration in TME. GSEA found that PTPRN influenced several immune-related pathways, suggesting that PTPRN might participate in EMT and regulated immune infiltration. The results of CIBERSORTx and GEPIA indicated that the expression of PTPRN is correlated with type markers of different immune cells, especially NK cells.

The antitumor function of innate immunity has gained great attention. NK cells, a subset of lymphoid cells triggering innate immunity, exert natural cytotoxicity against cancer cells and inhibit metastasis to different tissues (Wu et al., 2020). The main reason for cancer-associated deaths is metastasis, and immune escape leads to invasion-metastasis cascade (Lambert et al., 2017). NK cells which target tumor cells rapidly are suppressed by molecules derived from tumors, stromal cells educated by tumors, and cancer cells, leading to initiation and multiple metastatic processes of tumors (Wu et al., 2020). In our study, we found that the PTPRN high expression group expressed more NK cell inhibitory markers such as LIRB1 and KLRC1 in the TCGA-LUAD dataset. LDH assay indicated that A549 and H1299 cells with a PTPRN high expression level inhibit NK cell activation.

There are several strengths in our study. First, we found the impact of PTPRN in lung cancer, especially in LUAD. Second, we further identified the role of PTPRN in promoting migration and invasion of LUAD cells by regulating MEK/ERK and PI3K/AKT signaling pathways. Moreover, PTPRN was associated with immune cell infiltration in TME and inhibited NK cell activation. These results indicated that PTPRN could be a prognostic marker in LUAD patients and predict metastatic status of LUAD patients. PTPRN served as a potential antitumor immunotherapy strategy for LUAD patients. However, there are several limitations in our study. First, the way that PTPRN affected MEK/ERK and PI3K/AKT signaling pathways needed further study. Besides, biological mechanisms on how PTPRN influenced NK cell activation needed to be elucidated.

In conclusion, our study found that PTPRN can be a prognostic marker of LUAD patients and promotes the migration of LUAD cells *in vitro* and *in vivo*. Besides, PTPRN is correlated with immune infiltration of LUAD and inhibited NK cells activation. Our study identified the role of PTPRN in LUAD. Furthermore, PTPRN may play a key role in TME of LUAD by regulating immune cells infiltration especially NK cells, indicating that PTPRN as a potential and novel therapeutic target modulates antitumor immune response in treatment of LUAD.

## Conclusion

In conclusion, the study demonstrated that PTPRN was up-regulated in LUAD and promoted metastasis of LUAD patients. Furthermore, high PTPRN expression indicated poor OS in LUAD patients. Importantly, PTPRN promoted cell migration by regulating MEK/ERK and PI3K/AKT signaling pathways and influenced TIICs, especially inhibiting NK cell activation. The study reminds us that PTPRN might be a prognostic marker of LUAD patients and therapeutic target regulating antitumor immune response in LUAD treatment.

## Data Availability Statement

The original contributions presented in the study are included in the article/supplementary material, further inquiries can be directed to the corresponding author/s.

## Ethics Statement

The studies involving human participants were reviewed and approved by the study was supported by Ethics Committee of China Medical University and they claimed that they approved this study. All procedures performed in studies involving human participants were in accordance with the ethical standards of the institutional and national research committee and with the Helsinki declaration and its later amendments or comparable ethical standards. The plans for the use of the data were approved. The informed consent (written) is on file at China Medical University. The patients/participants provided their written informed consent to participate in this study. The animal study was reviewed and approved by all animal experiments were approved by the Institutional Animal Care and Use Committee of China Medical University.

## Author Contributions

XS, LZ, and MW conceived and designed the project. XS, XJ, MZ, and LJ designed and supervised experiments conducted in the laboratories. HY, LY, LC, and MJ performed experiments and/or data analyses. QW, LZ, and MW contributed reagents/analytic tools and/or grant support. XS and LW wrote the manuscript. XJ revised the manuscript. All authors discussed the results and commented on the manuscript.

## Conflict of Interest

MW was employed by Shenyang Kangwei Medical Laboratory Analysis Co. LTD. The remaining authors declare that the research was conducted in the absence of any commercial or financial relationships that could be construed as a potential conflict of interest.

## Footnotes

1 https://portal.gdc.cancer.gov/

2 http://kmplot.com/analysis/

3 https://cibersortx.stanford.edu/

4 http://gepia.cancer-pku.cn/

## Abbreviations

LUAD: Lung adenocarcinoma
PTPRN: Receptor-type tyrosine-protein phosphatase-like N
TCGA: The Cancer Genome Atlas
EMT: Epithelial–mesenchymal transition
NK cells: Natural killer cells
NSCLC: Non-small cell lung cancer
OS: Overall survival
TME: Tumor microenvironment
TIICs: Tumor-infiltrating immune cells
TAM: Tumor-associated macrophage
Th1 cells: T-helper 1 cells
Th2 cells: T-helper 2 cells
Tfh cells: Follicular helper T cells
Th17 cells: T-helper 17 cells
Treg cells: Regulatory T cells
GSEA: Gene-set enrichment analysis
PBMC: Peripheral blood mononuclear cells
LDH: Lactate dehydrogenase
ELISA: Enzyme-linked immunosorbent assay
LLC: Lewis lung carcinoma
IHC: Immunohistochemistry.
